# Myoclonic Epilepsy With Ragged Red Fiber Cardiomyopathy: A Case Report and Brief Review of Literature

**DOI:** 10.7759/cureus.66745

**Published:** 2024-08-13

**Authors:** Alessandro Giaj Levra, Francesco Amata

**Affiliations:** 1 Department of Biomedical Sciences, Humanitas University, Pieve Emanuele, ITA; 2 Cardio Center, Scientific Institute for Research, Hospitalization and Healthcare (IRCCS) Humanitas Research Hospital, Rozzano, ITA

**Keywords:** mitochondrial disease, hypertrophic cardiomyopathy, merrf, dilated cardiomyopathy, heart failure

## Abstract

Myoclonic epilepsy with ragged red fibers (MERRF) is a rare mitochondrial disease that can affect various organs, including the heart. We present a case report and brief review of the literature with the aim of exploring the progression of cardiac involvement in patients with MERRF.

A 65-year-old male with a history of MERRF, first diagnosed at age 55 with interventricular septum hypertrophy, presented with acute heart failure. The patient’s clinical course over 10 years demonstrated a transition from a hypertrophic to a dilated cardiomyopathy phenotype, contrasting earlier findings suggesting rapid progression in younger patients. Despite optimized heart failure therapy, the patient experienced a progressive decline in ventricular function with frequent ventricular arrhythmias, ultimately requiring implantable cardioverter-defibrillator (ICD) placement.

This case supports the hypothesis that MERRF-related cardiac involvement may progress more slowly when onset occurs later in life, leading to a gradual transition from hypertrophic to dilated cardiomyopathy. An accurate cardiac diagnostic workup is essential for early detection and timely intervention in such patients.

The natural history of cardiac involvement in MERRF can vary significantly based on the age of onset, highlighting the importance of personalized diagnostic and therapeutic approaches in managing this rare mitochondrial disorder.

## Introduction

Myoclonic epilepsy with red ragged fibers (MERRF) is a rare mitochondrial disease, associated with the A-to-G mutation at nucleotide position 8344 of mitochondrial DNA (mtDNA) for transfer RNA of lysine. Typical phenotypes include the onset of myoclonic seizures, lipomatosis, eyelid ptosis, and hearing loss [1,2]. Cardiac involvement has been previously described and is usually associated with the development of arrythmias and hypertrophic or dilated cardiomyopathy (DCM) [3]. Typical onset of cardiac involvement in adult life has been reported between the third and fifth decades of life [4-6]. We report a case of a patient affected by MERRF who exhibited a delayed onset of heart failure (HF).

## Case presentation

A 65-year-old male presented to the emergency department (ED) with a two-week history of progressively worsening shortness of breath, cough, and frequent falls without loss of consciousness.

His past medical history included a diagnosis of MERRF at age 55, attributable to an A-to-G mutation at nucleotide position 8344. Particularly, genetic testing revealed a mutant mtDNA proportion of 50% in blood and 70% in urinary sediment. The patient reported persistent tremors in the head and upper limbs, hearing loss, exercise intolerance, and progressive cognitive impairment. Additionally, he experienced upper limb myoclonus, primarily occurring at night. Lower limb sensory and motor polyneuropathy had also been previously diagnosed. Transthoracic echocardiogram (TTE) at the time of diagnosis showed normal dimensions of the cardiac chambers with preserved left ventricular ejection fraction (LVEF) and slight interventricular septum hypertrophy (12 mm). Family history indicated that the patient's sister was also affected by the same mitochondrial disease, while his mother, who died of pancreatic cancer, had never undergone genetic testing. The patient did not take any medications and did not report follow-up thereafter.

**Figure 1:**
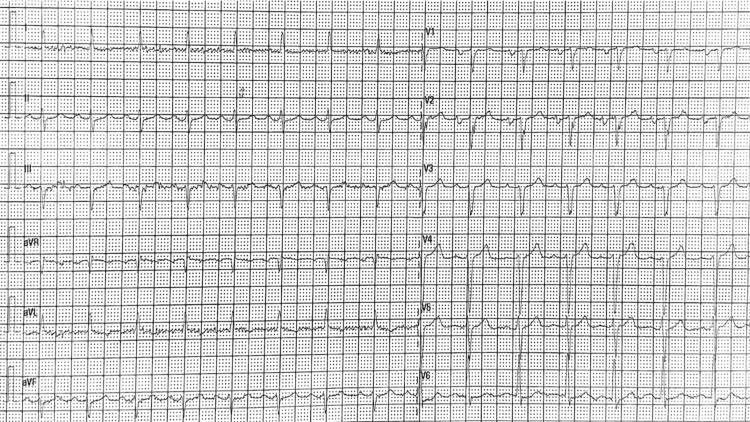
Electrocardiogram at admission showing a sinus rhythm with normal atrioventricular conduction, intraventricular conduction, and ventricular repolarization.

Upon admission to the ED, physical examination showed a systolic cardiac murmur, lower limb edema, pulmonary crepitations, hepatojugular reflex, and elevated jugular venous pressure. One lipoma was noted on the patient’s back. The patient was hemodynamically stable and had a good peripheral oxygen saturation. Electrocardiogram (ECG) showed sinus rhythm with normal atrioventricular and intraventricular conduction, as well as normal ventricular repolarization (Figure 1).

**Figure 2:**
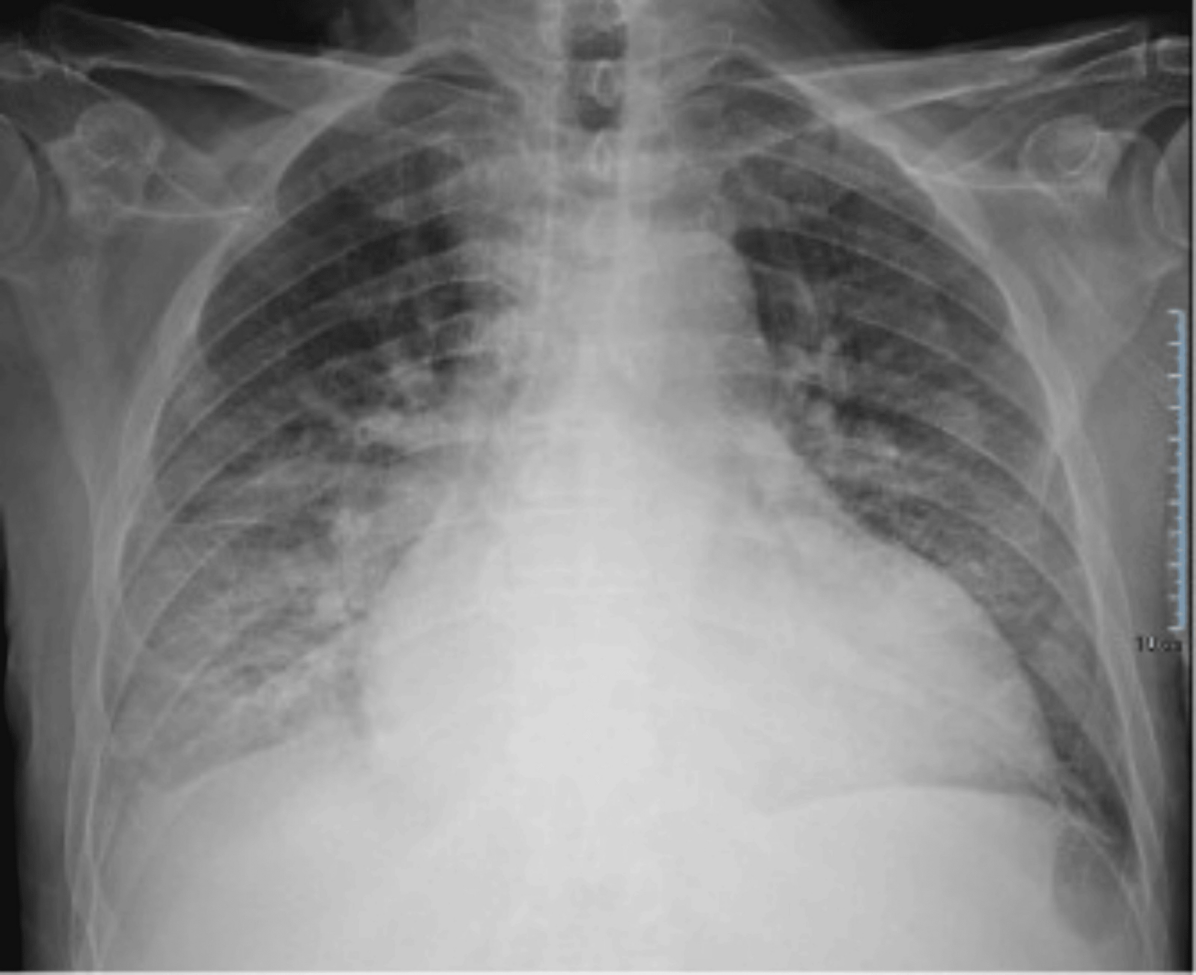
Chest X-ray at admission showing cardiomegaly with signs of pulmonary venous congestion.

Blood tests revealed elevated N-terminal pro b-type natriuretic peptide (NT-proBNP) levels (6,985 pg/mL) and a mild increase in high-sensitivity troponin T (hs-TnT) concentration (59 pg/mL). Blood cell count, electrolytes, and renal and liver functions were all within normal ranges, and inflammatory biomarkers were negative. Chest X-ray showed cardiomegaly and pulmonary venous congestion (Figure 2).

**Figure 3:**
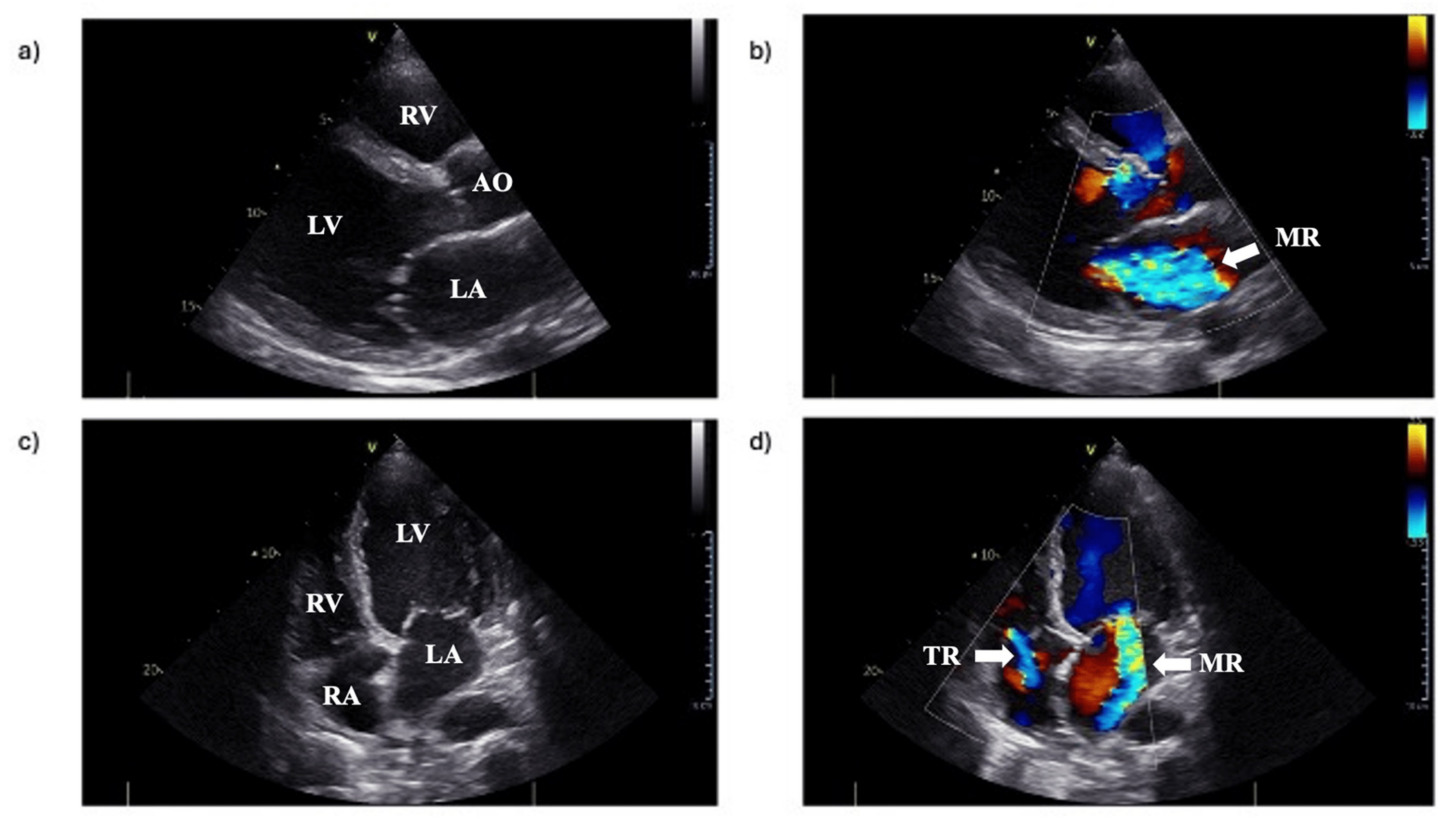
Transthoracic echocardiogram at admission showing a severely dilated left ventricle with severe mitral regurgitation and moderate tricuspid regurgitation. a) PLAX view. b) Color flow Doppler imaging on the PLAX view. c) AP4CH view. d) Color flow Doppler imaging on the AP4CH view. AO, aorta; AP4CH, apical four-chamber; LA, left atrium; LV, left ventricle; MR, mitral regurgitation; PLAX, parasternal long axis; RA, right atrium; RV, right ventricle; TR, tricuspid regurgitation

Acute decompensated HF was diagnosed; therefore, intravenous furosemide was started, and the patient was admitted in the cardiology ward. The diagnostic workup included a TTE that revealed a markedly dilated and diffusely hypokinetic left ventricle, with a severely reduced LVEF (30%). Interventricular septal thickness was normal (10 mm). Severe mitral regurgitation (MR) and moderate tricuspid regurgitation (TR) were present. Tricuspid annular plane systolic excursion (TAPSE) was 22 mm, indicating normal right ventricular longitudinal function. Inferior vena cava was dilated (24 mm) with no significant respiratory diameter variation (Figure 3).

**Figure 4:**
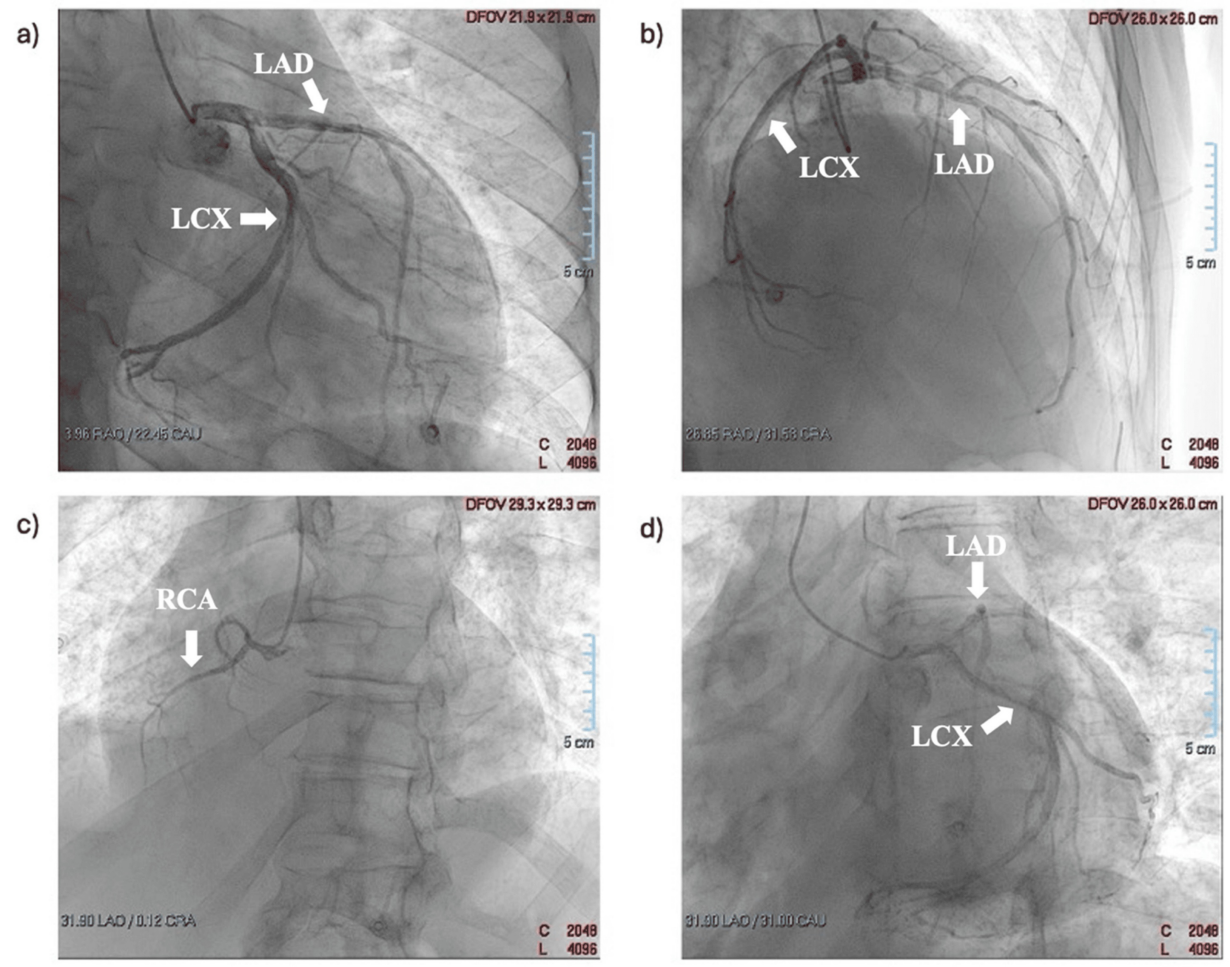
Coronary angiographic projections showing hypoplasia of the right coronary artery and the absence of hemodynamically significant coronary artery stenosis. a) Right caudal. b) Right cranial. c) Left cranial. d) Left caudal. LAD, left anterior descending; LCx, left circumflex; RCA, right coronary artery

To exclude a possible ischemic cause of left ventricular dysfunction, the patient also underwent coronary angiography, which revealed hypoplasia of the right coronary artery without any hemodynamically significant stenosis of the coronary arteries (Figure 4).

**Figure 5:**
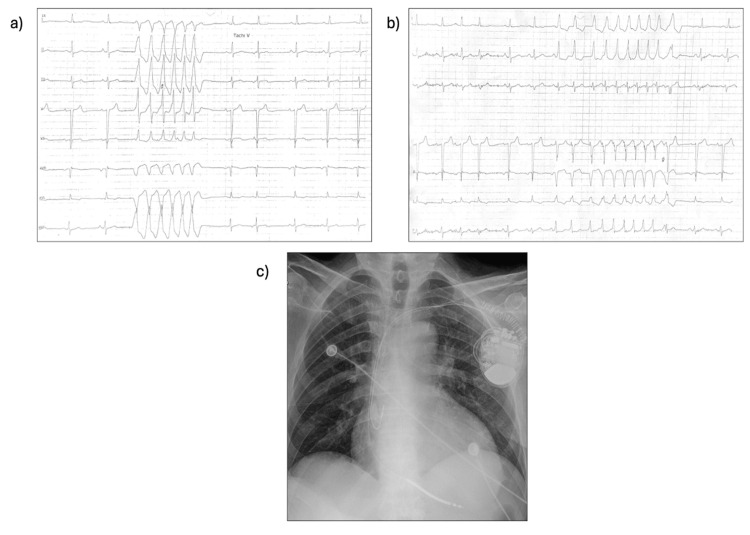
Examples of NSVT and post-operative chest X-ray a,b) Some examples of NSVT recorded during in-hospital ECG monitoring. c) Chest X-ray control after ICD placement. ICD, implantable cardioverter-defibrillator; NSVT, non-sustained ventricular tachycardia

Cardiac magnetic resonance imaging (CMR) was considered, but this diagnostic step was omitted due to the patient’s tremors and difficulty lying still in an enclosed space. After ruling out major causes of left ventricular dilation and dysfunction, the final diagnosis was DCM secondary to MERRF. The patient initiated pharmacological treatment for HF, including sacubitril/valsartan, beta-blocker, spironolactone, and sodium-glucose cotransporter-2 (SGLT2) inhibitor, along with furosemide, leading to a gradual resolution of congestive signs and symptoms. During hospitalization, ECG monitoring recorded multiple episodes of non-sustained ventricular tachycardia (NSVT) that recurred despite antiarrhythmic therapy and the resolution of congestion. Therefore, the patient was considered to be at increased arrhythmic risk and underwent implantable cardioverter-defibrillator (ICD) implantation (Figure 5).

The patient was discharged after 20 days of hospitalization with optimized medical therapy for HF, alongside a physical therapy regimen, a home assistance program, genetic counselling, and scheduled follow-up at the HF clinic. Pre-discharge TTE showed a persistent dilation and severe dysfunction of the left ventricle, and a slight improvement in the severity of valve regurgitations, with residual moderate MR and mild TR. Transmission from the ICD a month after the discharge showed a stable rhythm with no defibrillations delivered.

## Discussion

The present report describes a case of acute decompensated HF with reduced LVEF secondary to a MERRF-related DCM. Little is known about the optimal management of cardiomyopathies secondary to mitochondrial diseases [7,8]. Current therapeutical recommendations are independent from underlying etiologies of DCM [9]. Catteruccia et al. reported poor response to HF therapy and progressive decline in ventricular function in one patient who developed the DCM phenotype, ultimately requiring ICD implantation at the age of 60 [10].

Wolff-Parkinson-White syndrome has been reported as the most common arrythmia associated with MERRF [3]. However, Wet al.et al. underlined the increased risk of arrythmias in patients with mitochondrial dysfunction [11]. Furthermore, the development of myocardial dysfunction in patients with MERRF was associated with an increased risk of sudden cardiac death [12]. Therefore, our patient was considered as having an increased arrhythmic risk, and ICD implantation was performed to protect the patient from future life-threatening arrhythmias. Anan et al. previously described the cardiac phenotype of MERRF. In their study, patients were initially characterized by asymmetric septal hypertrophy that evolved into DCM. They hypothesized that gradual mitochondrial accumulation may drive the progressive myocardial changes. Similarly, our patient had evidence of previous septal hypertrophy with preserved LVEF that evolved into a dilated left ventricular cavity with a reduced LVEF. Differently, the progression of disease in their study was of two years, whereas our patient developed a dilated left ventricle after 10 years without receiving any medical therapy [3]. A possible explanation may reside in the poor adherence to follow-up. Gradual changes in the left ventricle may have gone unseen, resulting in an acute HF episode 10 years later, thus underlying the importance of cardiac monitoring in patients with A-to-G mutation at nucleotide position 8344 in MERRF syndrome.

Morgan et al. reported a case of a 33-year-old male affected by a mitochondrial disease that rapidly developed HF and required heart transplantation. Surgical specimen demonstrated dilated ventricles with scattered hypertrophic cardiomyocytes, further supporting the hypothesis of Anan et al. [13]. In our patient, dilation was limited to the left ventricle with no sign of right-sided dysfunction. Possibly, right ventricular dysfunction may represent the next step in disease progression.

Florian et al. performed CMR in 64 mitochondrial myopathy patients, three of which had MERRF and had a mean age of 46 years. CMR showed mild reduction in LVEF and mild septal hypertrophy in the absence of late gadolinium enhancement (LGE) [14]. Catteruccia et al. instead reported non-ischemic LGE pattern in the infero-lateral segments [10]. CMR was not feasible in our patient due to motor symptoms of the condition, and LGE assessment could have further defined the arrhythmic risk and better defined the myocardial dysfunction.

Mitochondrial disorders are known to have a wide phenotypic spectrum. In the present report, the patient exhibited milder neurological involvement compared to cases present in the literature [15]. Cardiac involvement appears to be rare in mitochondrial diseases with predominant neurological symptoms [5]. Conversely, A-to-G mutation at position 8344 seemed to prevalently affect muscle tissue with mild central nervous system involvement [10]. Indeed, our patient reported myoclonic spasms but no evidence of seizures at previous EEG, motor tics, and hearing loss. Slight cognitive decline was noted in 2017, but the patient maintained good autonomy in daily life activities up to the time of hospitalization.

## Conclusions

MERRF is a rare mitochondrial disease that can affect the cardiovascular system. This case report supports the hypothesis that the natural history of MERRF involves a gradual progression from a hypertrophic phenotype to a dilated form over several years. An accurate cardiac diagnostic workup is crucial in managing these patients, as it allows for the early detection of HF signs and the prompt initiation of medical therapy.
